# Molecular chaperone accumulation as a function of stress evidences adaptation to high hydrostatic pressure in the piezophilic archaeon *Thermococcus barophilus*

**DOI:** 10.1038/srep29483

**Published:** 2016-07-05

**Authors:** Anaïs Cario, Mohamed Jebbar, Axel Thiel, Nelly Kervarec, Phil M. Oger

**Affiliations:** 1 Univ Lyon, ENS de Lyon, CNRS UMR 5276, Lyon, France; 2 Univ Brest, CNRS, Ifremer, UMR 6197-Laboratoire de Microbiologie des Environnements Extrêmes (LM2E), Institut Universitaire Européen de la Mer (IUEM), rue Dumont d’Urville, 29 280 Plouzané, France; 3 Univ Brest, PLATE-FORME TECHNOLOGIQUE RMN-RPE-SM, UFR Sciences et Techniques, Avenue Le Gorgeu, Brest, France; 4 Univ Lyon, INSA de Lyon, CNRS UMR 5240, Lyon, France

## Abstract

The accumulation of mannosyl-glycerate (MG), the salinity stress response osmolyte of *Thermococcales*, was investigated as a function of hydrostatic pressure in *Thermococcus barophilus* strain MP, a hyperthermophilic, piezophilic archaeon isolated from the Snake Pit site (MAR), which grows optimally at 40 MPa. Strain MP accumulated MG primarily in response to salinity stress, but in contrast to other *Thermococcales*, MG was also accumulated in response to thermal stress. MG accumulation peaked for combined stresses. The accumulation of MG was drastically increased under sub-optimal hydrostatic pressure conditions, demonstrating that low pressure is perceived as a stress in this piezophile, and that the proteome of *T. barophilus* is low-pressure sensitive. MG accumulation was strongly reduced under supra-optimal pressure conditions clearly demonstrating the structural adaptation of this proteome to high hydrostatic pressure. The lack of MG synthesis only slightly altered the growth characteristics of two different MG synthesis deletion mutants. No shift to other osmolytes was observed. Altogether our observations suggest that the salinity stress response in *T. barophilus* is not essential and may be under negative selective pressure, similarly to what has been observed for its thermal stress response.

It is now well established that the majority of life on Earth dwells in the depth, in the so-called “Deep-Biosphere”, although due to the difficulties accessing those there is only scarce information on how the ecosystems function. Amongst deep-biosphere ecosystems, hydrothermal vents are one of the most intriguing. Hydrothermal vent fluids form when seawater enters cracks in the substratum near spreading ridges. During its interaction with the warm basalt, the water gets saturated with minerals, which composition will vary with the substratum^1^. Hydrothermal fluids are eventually forced out from the seafloor. Upon release in the ocean, the warm fluid gets into contact with the cold, oxidized ocean waters, creating a very sharp gradient in temperature, salinity, and redox potential over a few centimeters^2^,^3^,^4^. Despite these extremely variable environmental conditions, microbial life is thriving in the hydrothermal fluids. Thus, it is expected that microorganisms from hydrothermal vents express a strong stress adaptation in order to live in these steep gradients.

The effects of salinity and thermal stresses on cells and biological macromolecules are essentially negative, e.g. destabilization of the function and structure of cellular components or inward/outward fluxes of cellular salts. Two strategies of osmoadaptation to salinity have been demonstrated in prokaryotes^5^. Extremely halophilic *Archaea* and a few halophilic *Bacteria* accumulate K^+^, Na^+^ and Cl^−^ in response to changes in extracellular salinity^6^,^7^,^8^, while a common strategy among microorganisms to cope with osmotic stress will involve the accumulation of low-molecular-mass organic compounds, also named compatible solutes because they do not interfere with cellular metabolism^9^,^10^,^11^. Compatible solutes can be sugars, amino acids, polyols and are shared between thermophiles and mesophiles. Hyperthermophiles and thermophiles also accumulate very specific solutes little or never encountered in mesophiles: mannosylglycerate (MG), which is accumulated by hyperthermophilic *Archaea* and thermophilic *Bacteria*^12^; di-*myo*-1,3′-inositol phosphate (DIP)^13^, which is restricted to the domain *Archaea* with the exception of two hyperthermophilic bacterial genera, i.e. *Thermotogales* and *Aquificales*^14^,^15^,^16^,^17^; diglycerol phosphate and derivatives of these compounds^5^,^18^. MG is accumulated in response to high salinity. Cellular concentrations exceed 0.6 μmol/mg of proteins in *Thermococcales*^12^,^19^,^20^,^21^. MG is synthesized in four steps from mannose-6P by proteins encoded by a four-gene cluster which is strongly conserved in the *Thermococcales*, to the exception of the *T. kodakarensis* species which genome lacks the entire locus (Fig. 1). DIP accumulates in response to high temperature stress to cellular concentrations in excess of 1 μmol/mg of proteins^12^,^21^. Thus, MG and DIP have been proposed to be key players in osmo- and thermo-protection in hyperthermophiles respectively^12^,^22^.

In the deepest parts of the oceans, hydrothermal vent ecosystems are also submitted to extremely high hydrostatic pressures (HHP)^23^,^24^. Several microbial species adapted to extreme pressures (piezophiles) have been isolated from various deep-sea hydrothermal vents over the last decades^2^,^25^,^26^,^27^,^28^. The first piezophilic hyperthermophilic isolate was *Thermococcus barophilus* strain MP, isolated from the Snake Pit hydrothermal vent system (3,550 m depth) on the Mid-Atlantic Ridge, which grows optimally at 40 MPa, 85 °C and 3% salinity^26^. More recently, *Pyrococcus yayanosii* strain CH1, an obligate piezophilic hyperthermophile was isolated from the Ashadze hydrothermal vent site, also on the Mid-Atlantic Ridge^27^,^29^. To date, the genetic bases of the adaptation to HHP in these piezophiles remain unknown^30^,^31^.

The impact of pressure bears resemblance to both a lowering of temperature and an increase in temperature, since it will reinforce the structure of some molecules, such as membrane lipids, but will as well destabilize other structures, such as proteins^30^,^32^,^33^. In some ways, it also bears resemblance to salinity stress. Since HHP will antagonize or emphasize the impact of thermal and salinity stresses on cells, it has been proposed that HHP may influence the cellular response to stress in piezophiles^34^,^35^,^36^. The presence of DIP and MG synthesis genes in the genomes of the two known hyperthermophilic piezophiles, *T. barophilus* and *P. yayanosii*^37^,^38^, suggests that the stress response in these organisms could rely on the same bases as in other *Thermococcales*. However, recent investigations of the thermal stress response in *T. barophilus* showed that this strain is unable to synthesize DIP, due to a four-gene insertion between the IPS and IMPCT/PIPS genes^36^. These genes code for an Inositol-1-phosphate synthase and a bifunctional CTP:Inositol-1- phosphate cytidylyltransferase/Phospho-di-inositol-1-phosphate synthase, respectively responsible for the first two steps of DIP synthesis. The MG synthesis cluster in *T. barophilus* is also interrupted by a 17kb insertion between the MPGS and MPGP encoding genes (Fig. 1), which might also significantly affect the expression of MG synthesis genes, and consequently the salinity stress response of *T. barophilus*. These two genes encode the proteins responsible for the last two steps of MG synthesis^20^. Thus, whether MG is accumulated in *T. barophilus* in response to salinity stress, thermal stress, or both stresses as has been observed in *P. ferrophilus*, remained to be determined.

To address this question, we have investigated the effect of HHP on the salinity and thermal stress response of *Thermococcus barophilus* strain MP^26^. We show here that MG is accumulated primarily in response to salinity stress in *T. barophilus*. Stress response and the accumulation of MG peaked at low pressure, which clearly demonstrates the protective effect of HHP against salt and thermal stresses and a physiological adaptation to HHP of the *T. barophilus* proteome.

## Results

### Characterization of the response to salt and heat stress in *T. barophilus*

Salinity, and combined thermal/salinity/pressure stress conditions for *T. barophilus* were optimized in order to achieve comparable growth parameters under both sub-optimal and supra-optimal conditions. The detailed procedure and results are described in the Supplementary Material (Table S1). Our results show that the high hydrostatic pressure had no impact on the optimal temperature or salinity for *T. barophilus*. Sub-optimal and supra-optimal salinity stresses were 1% and 4% salinity. These conditions resulted in a 12 h growth lag in cultures but had little impact on the final growth yields. Organic solutes were extracted from *T. barophilus* cells grown under sub-optimal, optimal and supra-optimal conditions in temperature (80 °C, 85 °C, 90 °C respectively), salinity (1%, 3%, 4% NaCl) and hydrostatic pressure (0.1 MPa, 40 MPa, 70 MPa), representing a total of 27 different growth conditions. The ethanol extracts were examined by natural abundance ^1^H- and ^13^C-NMR for the presence of MG. The ^1^H- and ^13^C-NMR spectra showed peaks corresponding to α-mannosyl-glycerate, easily identified by comparison with those of the literature^39^. The specific assignments of MG are reported on the ^1^H-^13^C HMQC spectra (Figure S1). 2D-NMR (HMBC and HMQC) spectra were acquired to confirm the structure of the putative MG present in *T. barophilus* extracts. The ^1^H-^13^C HMQC and HMBC spectra revealed the shift of the anomeric carbon (^1^H resonance at 4.92 ppm and ^13^C resonance at 100.1 ppm) which confirms the identification of this compound as MG. As expected from previous studies^36^, DIP was detected under none of the conditions tested in this study.

### Quantification of MG accumulation at optimal pressure under salinity and thermal stresses

Under optimal pressure and temperature growth conditions, i.e. 40 MPa and 85 °C respectively, MG was detected only for supra optimal salinities, e.g. 4 and 5% NaCl (Fig. 2, green rectangle). The absolute concentration of MG in cells reached 0.12 μmol/mg proteins for both conditions. At optimal pressure and salinity (Fig. 2, red rectangle), MG was not detected under standard thermal stress conditions (90 °C^36^). Under thermal stress conditions corresponding to the upper growth temperature limit for that species, e.g. 98 °C, MG accumulation was detected at very low levels (0.06 μmol/mg proteins). When grown under both thermal and salinity stresses, MG was detected only under high salinity stress whatever the temperature tested. Its production peaked at 0.33 μmol/mg of proteins for a NaCl concentration of 4% and a temperature of 90 °C. Temperature higher than 90 °C could not be tested at or above 4% salinity for lack of growth of strain MP.

### Effect of hydrostatic pressure on the salinity and thermal stress response in *T. barophilus*

Under sub-optimal pressure conditions and under optimal salinity and temperature conditions (85 °C, 3%NaCl, 0.1 MPa, Fig. 2), MG is accumulated at 0.30 μmol/mg of proteins, a level comparable to the highest MG accumulation observed under optimal growth pressure. The salt stress response in *T. barophilus* at low pressure is similar whatever the temperature tested. No MG was detected at low salinity; while the MG accumulated at 4% NaCl was almost two folds that accumulated at “optimal” salinity. MG accumulation peaked for the combined high salinity and high temperature stress to reach 0.6 μmol/mg of proteins.

Under supra-optimal pressure conditions, MG was almost never detected in the cells. In fact, MG was accumulated to levels close to the detection limit between 0.024 and 0.03 μmol/mg of proteins, only under high salt stress. Due to the very limited amount of MG accumulated at supra-optimal pressure, it is difficult to ascertain whether its accumulation in the cells of *T. barophilus* followed the same trend at supra-optimal pressure as is observed for the other two pressure conditions, although the detection of MG only under the conditions yielding the highest accumulation at sub-optimal and optimal pressures would be consistent with such a scenario. Noticeably however, there is a marked negative correlation between pressure and MG accumulation in response to salt and heat stresses in *T. barophilus*.

### Salinity and thermal stress response of MG-deficient mutants of *T. barophilus*

**Figure 1:**
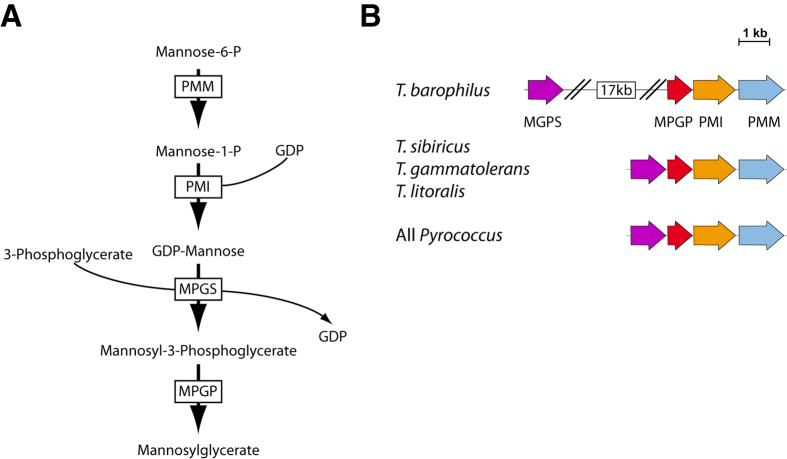
MG synthesis pathway (**A**, adapted from ref. 20) and genetic organization (**B**).

**Figure 2:**
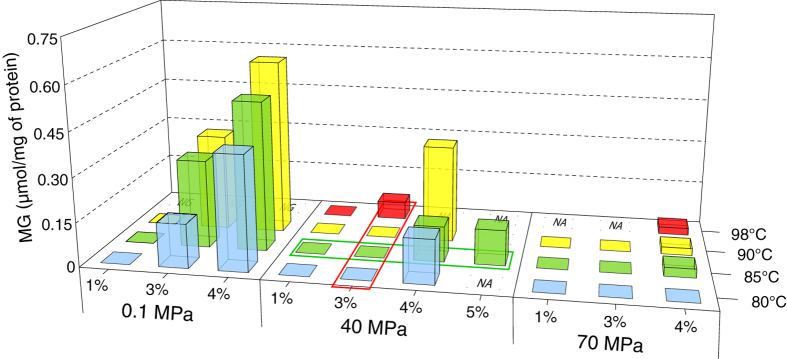
MG accumulation in *T. barophilus* as a function of pressure, in response to salt and temperature stress. Thermal stress conditions under optimal salinity (3% NaCl) and pressure (40 MPa) conditions for growth are highlighted by a red rectangle. Salinity stress conditions under optimal temperature (85 °C) and pressure (40 MPa) are highlighted by a green rectangle. NG (No growth) P/T/salinity combination incompatible with growth of *T. barophilus*, e.g. 98 °C at atmospheric pressure. NA: Not analyzed.

Regardless of the accumulation pattern of MG, which exhibits a typical stress response, the absolute levels of MG accumulated in the cells remained low in comparison to other *Thermococcales*, raising questions about the role of MG and other organic solutes in the stress response of *T. barophilus*. To understand the contribution of MG in salt or thermal adaptation, two mutants lacking MG synthesis were constructed in the ∆*pyrF* derivative of *T. barophilus*^40^. In Tb∆MGPS, MGPS, the isolated gene of the pathway, which encodes the third enzyme of the MG pathway catalyzing the conversion of GDP-mannose to mannosyl-3-phosphoglycerate, was deleted (Fig. 1); while in Tb∆MPGP the first gene of the operon, which encodes the last enzyme of the MG synthesis pathway catalyzing the conversion of mannosyl-3-phosphoglycerate to mannosylglycerate, was deleted. Both mutants showed lower growth rates under combined salinity and thermal stress conditions (90 °C and 4% NaCl) at 0.1 MPa, while the Tb∆MPGP mutant also showed reduced growth under optimal growth conditions (Table 1). Growth yields were essentially unaffected at 24 or 48 h (Table S2), although some small but reproducible differences could be observed when the cells were subcultured from sub-optimal salinity to supra-optimal salinity stress conditions (Table S3). As expected, no MG synthesis could be detected in the two mutants under any of the pressure, temperature and salinity combinations tested, while MG synthesis could be detected at wild-type levels in the ∆*pyrF* parent strain. In this later strain, MG accumulation peaked at similar concentration for combined temperature and salinity stress (0.59 and 0.62 μmol/mg of proteins, respectively). Interestingly, the accumulation of the last intermediate in MG synthesis, e.g. mannosyl-3-phosphoglycerate, was detected at a very low level (0.0011 μmol/mg of proteins) in the Tb∆MPGP mutant for combined temperature and salinity stress.

### Accumulation of compatible solutes in wild-type and MG-deficient mutants of *T. barophilus*

Aspartate and glutamate have been previously shown to accumulate in *Thermococcales* to compensate for the absence of DIP synthesis^41^,^42^. To evaluate the possibility of cross compensation of the lack of MG accumulation by other organic solutes, we quantified the accumulation of aspartate and glutamate in *T. barophilus* cells. In the wild-type *T. barophilus* cells grown under optimal conditions, aspartate, glutamate and total organic solutes were present at 0.07, 0.14 and 0.31 μmol/mg of proteins respectively (Table 2). Variations of solutes concentrations under stress conditions are quite small. The total solute pool increases almost two times between optimal and combined salinity and thermal stresses. This increase is essentially due to the accumulation of MG, although aspartate accumulation is decreased slightly under thermal stress. In the Tb∆MPGP mutant, aspartate and glutamate concentration variations are also small. Aspartate concentrations decrease by a factor close to 2 under salinity stress, while glutamate concentrations tend to increase. As a consequence, the total pool of organic solutes remains stable under stress. In contrast, aspartate, glutamate and total solute concentrations decreased under stress in the Tb∆MGPS mutant (Table 2). Intracellular K^+^ content was determined for all pressure, temperature and salinity conditions of growth in *T. barophilus* MP, yielding no evidence of inorganic solute accumulation as a function of stress in *T. barophilus* (Table S4).

## Discussion

We have monitored the accumulation of organic solutes in the piezophilic hyperthermophilic *Thermococcus barophilus* strain MP to characterize the impact of hydrostatic pressure and piezophilic adaptation on the salinity and thermal stress response in deep hydrothermal vent organisms. MG was the only osmolyte detected that responded to environmental stresses in *T. barophilus*. This solute is accumulated preferentially in response to salinity stress and its peak concentration is observed under combined salt, low-pressure and temperatures stresses. MG was shown to accumulate also under the extreme thermal stress conditions under optimal temperature and salinity. Interestingly, MG accumulation was twice higher under low hydrostatic pressure conditions in comparison to optimal pressure, while its accumulation was barely detected under supra optimal conditions. Osmolytes, such as MG or DIP, accumulate during stress to protect the proteome from the deleterious effect of the reduced activity of water inside or in the vicinity of the cells. The exact molecular mechanisms for osmolyte is still a subject of debate, but it has been proposed that osmolytes accumulated during stress create a protective shell surrounding the proteins, which helps maintain proper folding and protein function^43^. An increase of MG accumulation under low pressure conditions clearly indicates that low pressure are perceived as stressful conditions by the cell proteins and that the stability of its proteome is compromised, e.g. that the *T. barophilus* proteome is low-pressure sensitive. These results confirm and extend previous observations which have shown the pressure-dependent induction of a heat shock protein in strain MP^44^.

In a simplistic scheme, the impact of hydrostatic pressure may be resumed to the Le Chatelier general law of chemical equilibrium, which implies that an increase in pressure will favor the smallest state in a chemical system^45^. Thus, if the volume of a protein is smaller in its native form than in its unfolded form, this protein will be stabilized by pressure, and destabilized if its native volume is larger. Protein tightening under pressure is often reported^46^. Bartlett and colleagues have shown that enhanced pressure tolerance in the SSB protein in the piezophilic strain SS9 of *Photobacterium profundum* is linked to amino acid substitutions leading to increased rigidity of the protein structure^47^. This adaptation mechanism is essentially similar to the increased rigidification of proteins observed in the presence of the osmolyte MG^48^. Thus, the accumulation of MG under low pressure conditions will tend to increase protein rigidity, suggesting that the proteome of *T. barophilus* is too flexible at atmospheric pressure. In contrast, the lower accumulation of osmolytes under optimal and supra-optimal pressure conditions in *T. barophilus* suggests that high-hydrostatic pressure enhances protein stability in this organism. These observations are consistent with recent measurements of the molecular dynamics of *T. barophilus* cells by Neutron Scattering which have evidenced the extreme flexibility of the proteome of *T. barophilus* at atmospheric pressure and the stabilizing effect of hydrostatic pressure on the proteome^49^. These experiments clearly identified two dynamic regimes for the proteome of *T. barophilus*. In the first one, at atmospheric pressure the proteome flexibility is extremely high. Increased protein flexibility is an important part of the adaptation to low temperature in psychrophiles since it will counteract the temperature-dependent reduction of the dynamics. However, this increased flexibility is usually restricted to the active sites of the protein^50^,^51^,^52^ since an increase of the flexibility is linked to lower stability of the proteins. Indeed, an increase of the overall flexibility can have a deleterious impact on protein activity^53^,^54^,^55^,^56^. Molecular dynamics study in *T. barophilus* demonstrate an increase of the whole protein flexibility, not only of the actives sites, and suggests that it might impact significantly the proteome functions^49^. Consistent with this hypothesis, *T. barophilus* cells were shown to express a stress protein at ambient pressure^44^. The accumulation of MG, which has been shown to increase protein rigidity, at atmospheric pressure would also be consistent with the need for the cell to reduce this flexibility to restore the activity of the proteome^57^. In the second regime, which happens above 20 MPa, the flexibility of the proteome is greatly reduced and remains almost unaffected by pressure up to 120 MPa. This clearly shows the stabilizing impact of hydrostatic pressure on the proteome of *T. barophilus*, as well as the stability of this proteome under high hydrostatic pressures. This is congruent with the observation that MG is accumulated at lower levels, and that no stress protein are produced under pressure. Together, the physiological and molecular dynamics data concur to demonstrate that the proteome of *T. barophilus* is adapted to high hydrostatic pressure, e.g. piezophilic.

MG accumulation in *T. barophilus* at peak concentration under combined salt, low-pressure and temperature stresses remains in the low range (0.6 μmol/mg proteins) when compared to values reported for other *Thermococcales* under the sole salinity stress, e.g. from ca. 0.25 to more than 1 μmol/mg of proteins in *T. celer* and *P. furiosus* respectively^12^,^21^. Indeed, under similar stress conditions, MG accumulation is only 0.21 μmol/mg protein. Other solutes such as aspartate and glutamate may also play a role in the stress response of *Thermococcales*. Aspartate and glutamate levels were ca. 3-fold higher under osmotic stress in *T. litoralis*^21^, while in *T. kodakarensis*, glutamate accumulated 9.8-fold, concomitantly with a 20-fold DIP level increase, in response to heat stress, and aspartate concentrations increased 4.3 fold under osmotic stress^41^. In contrast to these *Thermococcales* species, the intra-cellular concentrations of aspartate and glutamate varied only marginally under thermal or salinity stress conditions in *T. barophilus* (Table 2). We could not detect significant organic solute concentration variations in the NMR spectra acquired in any stress conditions, suggesting that no other compatible organic solute was accumulated in this species in response to stress. Furthermore, we could not detect any organic osmolyte accumulation in two MG-deficient mutant derivatives of strain MP, and inorganic compatible solutes, such as K^+^ ions, which are accumulated in halophilic and slightly halophilic strains to counterbalance the negative charges in the cells^6^ are not accumulated in response to stress in *T. barophilus*. Thus, these results suggest that the salinity stress response is not essential in the *T. barophilus* species. Together with the loss of the thermal stress response which has been already reported in this species^36^, these results suggest that the *T. barophilus* species might have developed a novel strategy to respond to stress. However, these observations could also show that this species is losing its ability to respond to salinity and thermal stresses.

To this date, the DIP and MG loci and DIP and MG accumulation patterns were extremely conserved in all *Thermococcales*. Previous exceptions to this trend were *T. kodakarensis*, which lacks the MG synthesis locus; *Pyrolobus fumarii* which only accumulates DIP as a response to both stresses but which genome is yet uncharacterized^58^ and *Palaeococcus ferrophilus* which lacks the DIP synthesis genes and accumulates only MG as a response to both stresses. In contrast to these three exceptions, *T. barophilus* harbors a complete set of DIP and MG genes, but each is interrupted by a large insertion, e.g. ca 5 and 17kb for DIP and MG synthesis respectively (Fig. 1)^36^. These insertions are a reasonable explanation for the lack of DIP synthesis in *T. barophilus*^36^ but may also explain the low levels of MG accumulation. In contrast to other *Thermococcus* species, *T. barophilus* is piezophile, a phenotype shared with *P. ferrophilus* the other strain known not to accumulate DIP^19^,^59^. The lack of DIP accumulation in these two species was proposed to result from their adaptation to high hydrostatic pressures^36^. The rationale for this proposition relies on the overlaps existing between the impact of both stresses on proteins and on their antagonizing effects on biomolecules. Interestingly, the piezophilic *Thermotogales* species*, Marinitoga piezophila*, also lacks the DIP synthesis genes^60^ and accumulates only amino acids under tress conditions^17^. Thus, it is very tempting to try to link the low accumulation of organic solutes in *T. barophilus* and the piezophily of its proteome. The marginal impact of the disruption of MG synthesis in the MPGP and MGPS mutants is consistent with this view. Furthermore, and in contrast to other *Thermococcales*^41^, we observed no shift of the accumulation to other organic or inorganic solutes under stress, suggesting that stress response was not required for growth in *T. barophilus*. The observed accumulation of MG as a response to stress in wild-type *T. barophilus* may be a reminiscence of the salinity stress response of *Thermococcales* and an indication that MG has become non-essential only recently during the evolution of this species. Further experiments with DIP and MG synthesis knock-out mutants of other piezophilic hyperthermophiles, especially on the newly isolated obligate piezophile *Pyrococcus yayanosii* strain CH1, which cannot grow at atmospheric pressure^27^, would be required to confirm these hypotheses.

## Methods

### Microorganism and growth conditions

*Thermococcus barophilus* strain MP was grown in *Thermococcales* Rich Medium (TRM)^27^. The salinity of the medium was adjusted from 0 to 6% for salt-stress experiments. All cultures were performed under strict anaerobic conditions obtained by headspace substitution to N_2_ and by addition of Na_2_S.9H_2_O, pH = 7.2, to a final concentration of 0.1%. Cultivation at low pressure was performed in sealed serum vials while cultures under HHP were performed in sterile syringes as previously described^26^. Cultures were inoculated with 0.5% (v,v) of a glycerol stock, stored anaerobically at −80 °C at a starting cell concentration of 5.10^5^ cells per milliliter. To increase experimental reproducibility, the same inoculum was used for all cultures. Cultures were grown under 3 pressure conditions (0.1, 40 and 70 MPa), temperatures ranging from 75 to 98 °C and salinities ranging from 0 to 6% NaCl. Cell growth was monitored by direct cell counts in a Thoma chamber (depth, 0.01 mm) using a light microscope (BX41, Olympus) at regular intervals: 0, 12, 24, 48 and 72 h. Experiments were performed in triplicate unless specified otherwise.

### Extraction of intracellular solutes

10^10^ cells of *T. barophilus* were harvested during mid-exponential phase of growth by centrifugation (5,000 g, 15 min, 4 °C) and washed once with an isotonic NaCl solution. The washed cell pellets were extracted by the method of Reed^61^ except that the extraction was performed for 30 min in boiling 80% ethanol. Cells were removed by centrifugation 10 min at 12,000 g at 4 °C. Supernatants were transferred to a clean tube and dried in a rotary evaporator (IKA RV 10, Fisher Scientific, France) or lyophilized (Alpha 1–2 LDplus, Martin Christ, Germany). The dried residue was dissolved in D_2_O for further NMR analysis.

### NMR spectroscopy

All spectra were acquired on a Bruker DRX 500 spectrometer at room temperature and 30° pulse. ^1^H-NMR spectra were acquired using a 5 mm TBI ^1^H/{BB}/^13^C probe. Organic solutes were quantified by adding Trimethylsilyl-2,2,3,3-tetradeutero-propionic acid (TSP) as an internal standard. For quantification, spectra were acquired with water presaturation and a delay of 10s for T1 relaxation time. ^31^P-NMR spectra were acquired at 202.5 MHz on the same probehead. ^13^C-NMR spectra were acquired at 125.8 Mhz on a AVANCE 500 Bruker spectrometer equipped with a TCI ^1^H/^13^C/^15^N 5mm probehead. Two-dimensional spectra (COSY, HMQC and HMBC) were acquired with the same spectrometer. The values were normalized to the total protein content of cells quantified by the Bradford assay^62^ after cell lysis with 1 M NaOH (100 °C, 10 min) and neutralization with 1 M HCl^12^. Osmolyte concentrations are expressed as μmol solute/mg of total proteins.

### Extraction and determination of intracellular K^+^ content by ICP-AES

Cells of *T. barophilus* in mid-exponential phase were harvested by centrifugation (5,000 g, 15 min, 4 °C) and washed once in isotonic NaCl solution. Cell lysis was performed by boiling 30 min in distilled water. The supernatant was adjusted to 0.5 N HNO_3_ for inductively coupled plasma atomic emission spectrometry (ICP-AES) analysis. Calibration range was performed from 0 to 2 ppm. Scandium (1 ppm) was used as an internal standard.

### Construction of MG synthesis deletion mutants

Deletion mutants of the MGPS and were obtained as described^40^. The complete ORF of each gene was removed, to avoid polar effects on the downstream genes. The selection of mutants was performed under pressure, temperature and salinity conditions under which MG was not accumulated in the parental strain, e.g. low pressure, 85 °C and 1% NaCl. Proper deletion was confirmed by sequencing using primers located next to the deletion (Table S5).

## Additional Information

**How to cite this article**: Cario, A *et al*. Molecular chaperone accumulation as a function of stress evidences adaptation to high hydrostatic pressure in the piezophilic archaeon *Thermococcus barophilus*. *Sci. Rep*. **6**, 29483; doi: 10.1038/srep29483 (2016).

## Supplementary Material

Supplementary Information

## Figures and Tables

**Table 1:** Growth rate of wild-type *T. barophilus* (WT), Tb∆MPGP and Tb∆MPGS mutants at atmospheric pressure under optimal temperature and salinity conditions (85 °C and 3% NaCl), and temperature and salinity stress conditions (90 °C and 4% NaCl).

Conditions	Strains	μ (h^−1^)
3% NaCl/85 °C	WT	0.88 ± 0.08
	Tb∆MPGP	0.75 ± 0.08
	Tb∆MGPS	0.84 ± 0.09
4% NaCl/90 °C	WT	0.63 ± 0.21
	Tb∆MPGP	0.51 ± 0.12
	Tb∆MGPS	0.50 ± 0.12

Experiments were performed in quadruplicate.

**Table 2:** Quantitation of compatible solutes in *T. barophilus* MP and the Tb∆MPGP and Tb∆MGPS mutants.

Strain	Temperature	Salinity	Organic Solutes (μmol/mg protein)			
			MG	Asp	Glu	Total
MP	85 °C	3%	0,10	0.07	0.14	0.31
	85 °C	4%	0,21	0.11	0.29	0.61
	90 °C	3%	0,54	0.04	0.12	0.70
	90 °C	4%	0,62	0.07	0.14	0.84
Tb∆MPGP	85 °C	3%	ND	0.23	0.18	0.41
	85 °C	4%	ND	0.13	0.23	0.36
	90 °C	3%	ND	0.20	0.23	0.43
	90 °C	4%	ND*	0.16	0.24	0.40
Tb∆MGPS	85 °C	3%	ND	0.15	0.19	0.34
	85 °C	4%	ND	0.06	0.16	0.22
	90 °C	3%	ND	0.07	0.09	0.17
	90 °C	4%	ND	0.06	0.08	0.14

All clones were cultivated at 0.1 MPa. MG, mannosylglycerate; Asp, aspartate; Glu, glutamate.

^*^Mannosyl-3-phosphoglycerate, which is an intermediate of MG synthesis, was detected at a concentration of 0.0011 μmol/mg of proteins.
