# Supplementary material for: Molecular chaperone accumulation as a function of stress evidences adaptation to high hydrostatic pressure in the piezophilic archaeon Thermococcus barophilus
Source: Sci Rep. 2016 Jul 5;6:29483. doi: 10.1038/srep29483 (PMC4932500; doi:10.1038/srep29483)
Supplement: Supplementary Information [file srep29483-s1.pdf]

Molecular chaperone accumulation as a function of stress evidences adaptation to high hydrostatic pressure in the piezophilic archaeon *Thermococcus barophilus*

Anaïs Cario[1,+], Mohamed Jebbar[2], Axel Thiel[2], Nelly Kervarec[3], Phil M. Oger[1,4,*]

[1] Univ Lyon, ENS de Lyon, CNRS UMR 5276, Lyon (France)

[2] Univ Brest, CNRS, Ifremer, UMR 6197-Laboratoire de Microbiologie des Environnements Extrêmes (LM2E), Institut Universitaire Européen de la Mer (IUEM), rue Dumont d'Urville, 29 280 Plouzané (France)

[3] Laboratoire de RMN, Université de Bretagne Occidentale, UFR Sciences et Techniques, Avenue Le Gorgeu, Brest, (France)

[4] Univ Lyon, INSA de Lyon, CNRS UMR 5240, Lyon (France).

[+] Current address: Department of Earth and Environmental Sciences, Rensselaer Polytechnic Institute, Troy, New York 12180, USA

Running Title: Stress response proves piezoadaptation in *T. barophilus*

*** Corresponding author:** Phil M. Oger, PhD

Adaptation in Extreme Environments

UMR 5240 CNRS Microbiology, Adaptation and Pathogenicity

INSA de Lyon

Bâtiment Pasteur

11, Avenue Jean Capelle

F-69621 Villeurbanne cedex, France.

E-mail address: poger@insa-lyon.fr (P.M. Oger)

Supplemental data

Materials and Methods

RNA extraction, reverse transcription and quantitative PCR

Total RNAs were extracted from mid-exponential phase cultures following a single step RNA extraction procedure adapted from *Pyrococcus furiosus* [1,2]. The RNAs were treated with DNAse and further purified with the RNeasy kit from Qiagen according to the manufacturer's instructions. The absence of residual genomic DNA was verified by direct PCR amplification of 9 different gene targets (Table S6). Total RNA were reverse transcribed using the RevertAid™ H Minus Reverse Transcriptase kit (Fermentas, Lituany). The resulting cDNA was used as a template for target specific PCR amplification (95°C, 30s; 95°C, 30s, 56°C, 45s, 72°C, 45s, 39 cycles ; 72°C, 10mn) using primer pair for each MG and DIP genes from the *T. barophilus* genome (Table S6) [3]. Quantitative PCR assays were performed on a Mx3000 QPCR system (Agilent Technologies, Santa Clara, CA, USA) using the nonspecific fluorophore SYBR green I of the Brilliant II Ultra-Fast SYBR® Green QPCR master mix (Stratagene, La Jolla, CA, USA). The 20 µl reactions contained 1 µl of target cDNA (1 ng.µl^{-1}), 0.3 µl of 1/500 diluted reference dye and 1 µmol.l^{-1} of each forward and reverse primers. The PCR program consisted of 10 min at 95°C and 40 cycles of 95°C for 30 s, annealing at the specific primer hybridization temperature for 1 min, 72°C for 30 s to 1 min, and a final cycle of 1 min at 95°C, 30 s at 58°C and 30 s at 95°C. Melt curve analysis to detect the presence of primer dimers was performed after the final cycle by increasing the temperature from the hybridization temperature to 95°C in 0.5°C increments every 10 s. Negative controls without template were included in each run. A specific cloned reference was used for each target gene. Transcipts levels were normalized to 16S rRNA genes. Values are reported as a ratio of expression levels relative to optimal growth conditions (40MPa/85°C/3% NaCl).

Results

Influence of HHP on growth of *T. barophilus* under salinity stress and combined salinity and thermal stresses

To optimize salinity stress conditions, *T. barophilus* was grown in TRM at different salinities between 0% and 6% of NaCl under optimal pressure and temperature conditions, e.g 40 MPa and 85°C or varying temperatures ranging form 65°C to 98°C under optimal pressure and salinity conditions, e.g 40 MPa and 3% NaCl. As expected *T. barophilus* behaved as a slightly halophilic organism, requiring 1% - 5% NaCl in the culture medium [4]. Our results confirm that the highest growth rates and largest cell yields were obtained for cultures in 3% [4], the known optimal salinity for the strain. A 4h or 12h growth lag is observed in presence of 2% and 4% NaCl or 0, 1, 5 and 6% NaCl respectively (data not shown). A 0.3 log drop in growth yield is observed for 1, 4 and 5% salinities, while at the lowest (0%) and highest (6%) NaCl concentrations, almost no growth was observed. Based on these results, 1% and 4% NaCl were chosen as low and high saline stress conditions for further studies. Thermal stress conditions were similar to previous studies[5]. The supra-optimal pressure conditions were fixed at 70MPa, which was previously shown to match growth parameters for strain MP at 0.1MPa[6].

Growth yields were measured at 24h for sub-optimal, optimal and supra-optimal pressure, temperature and salinity conditions (Table S1). Our results show that hydrostatic pressure did not impact the optimal salinity conditions for *T. barophilus,* similarly to what was previously observed for temperature (Cario *et al.* 2015). As observed under optimal pressure conditions, temperature did not have a strong impact on growth at optimal salinity, while salinity greatly impacts cell growth under optimal temperature conditions (Table S1). The lowest growth yields are observed under low pressure conditions and either high or low salinity. Noticeably, growth yields under low or high salinity stress conditions were higher for the highest temperature.

Expression of MG synthesis genes in *T. barophilus*

The absence of DIP accumulation in *T. barophilus* cells grown under any of the growth conditions tested, correlated with the presence its genome of the three DIP synthesis genes (Figure 1) prompted us to explore the expression of the genes responsible for the synthesis of MG. Total RNAs were prepared in triplicate from cells grown under optimal conditions (40MPa, 85°C, 3%NaCl), under thermal stress (40MPa, 98°C, 3%NaCl) and conjugated salt and thermal stresses at low pressure (0.1MPa, 90°C, 4%NaCl). The expression of all four genes from the MG synthesis pathway could be easily detected under the three conditions tested. The PMI and PMM genes which encode the first two enzymes of the MG synthesis pathway were slightly over expressed under salt stress, while all four genes were over-expressed under thermal stress (Table S7).

Intracellular K^+ content as a function of salt and temperature stress

Intracellular K^+ content was determined for all pressure, temperature and salinity conditions of growth in *T. barophilus* MP and expressed relative to the value measured for optimal growth conditions, e.g. 201,6mM at 40 MPa, 85°C, 3% NaCl (Table S4). The values measured for intracellular K^+ ranged from 20,16 mM to 379 mM (0,1 MPa, 85°C, 4% NaCl and 40 MPa, 80°C, 1% NaCl, respectively). Significant intracellular K^+ accumulation was only observed in one condition under low salt and temperature at optimal pressure. Intracellular K^+ level was generally low, especially under salt stress conditions.

Pressure	0%	1%	2%	3%	4%	5%	6%

	80°C		7 ± 0.4	8 ± 0.1	7.9 ± 0.0	6.8 ± 0.3	6 ± 0.1	
0.1 MPa	85°C	6.1 ± 0.2	7.1 ± 0.2	8 ± 0.0	8 ± 0.1	6.8 ± 0.1	6.3 ± 0.2	6.2 ± 0.2
	90°C		7.6 ± 0.3	7.9 ± 0.1	7.9 ± 0.0	7.7 ± 0.1	6.2 ± 0.2	
	80°C		7.5 ± 0.1	8.1 ± 0.1	8.2 ± 0.1	7.8 ± 0.0	6.6 ± 0.1	
40 MPa	85°C	6.6 ± 0.3	7.7 ± 0.1	8.1 ± 0.1	8.3 ± 0.1	7.9 ± 0.0	7.3 ± 0.2	6.8 ± 0.2
	90°C		7.8 ± 0.2	8.1 ± 0.1	8.2 ± 0.1	8 ± 0.2	7.4 ± 0.4	
	80°C		7.6 ± 0.1	7.9 ± 0.2	7.9 ± 0.1	7.2 ± 0.6	6.1 ± 0.1	
70 MPa	85°C	5.8 ± 0.1	7.6 ± 0.1	8 ± 0.1	8 ± 0.1	7.7 ± 0.1	6.7 ± 0.1	6 ± 0.3
	90°C		7.7 ± 0.1	8 ± 0.2	8 ± 0.1	7.8 ± 0.1	6.7 ± 0.8	

Table S1: Growth yields after 24h of incubation under combined temperature, salinity and hydrostatic pressure stress expressed in log of cells per ml.

**Time (h)**	**Salt growth contitions**	**Delta log number of cells/ml**			
		**MP**	***deltapyrf***	***deltampgp***	***deltamgps***
**8**	1% NaCl	1.3 ± 0.14	1.1 ± 0.25	1.3 ± 0.27	1.0 ± 0.10
	3% NaCl	2.1 ± 0.18	2.0 ± 0.09	1.9 ± 0.28	2.0 ± 0.21
	4% NaCl	0.6 ± 0.09	0.6 ± 0.06	0.7 ± 0.02	0.7 ± 0.03
**24**	1% NaCl	2.2 ± 0.01	2.0 ± 0.11	2.1 ± 0.16	2.1 ± 0.12
	3% NaCl	2.4 ± 0.17	2.2 ± 0.02	2.3 ± 0.28	2.4 ± 0.22
	4% NaCl	0.9 ± 0.14	1.3 ± 0.20	1.2 ± 0.14	1.2 ± 0.23
**48**	1% NaCl	2.2 ± 0.09	2.0 ± 0.03	2.2 ± 0.13	2.2 ± 0.23
	3% NaCl	2.4 ± 0.10	2.2 ± 0.11	2.3 ± 0.27	2.4 ± 0.16
	4% NaCl	2.4 ± 0.18	2.1 ± 0.11	2.2 ± 0.26	2.3 ± 0.27

Table S2 : Growth yield after 8, 24 and 48h of *T. barophilus* strain MP (parental), TbΔpyrF, TbΔMPGP and TbΔMGPS mutants cultivated at atmospheric pressure and 85°C at different salinities (1, 3 and 4% NaCl) from a pre-culture grown at atmospheric pressure, 85°C and 1% NaCl. Values are an average of triplicate experiments.

T. barophilus	**Pressure**	**0.1 MPa**			**40 MPa**		**70 MPa**
strains	**Salinity**	**1%**	**3%**	**4%**	**3%**	**4%**	**3%**
**WT**		8.10	8.45	8.26	8.52	NA	8,28
**TbΔMPGP**	**85°C**	8.11	8.40	8.08	8.45	NA	7,99
**TbΔMGPS**		8.08	8.38	8.08	8.41	NA	7,99
**WT**		7.95	8.26	8.32	NA	8.34	NA
**TbΔMPGP**	**90°C**	7.56	8.26	8.26	NA	8.11	NA
**TbΔMGPS**		8.04	8.30	8.15	NA	8.08	NA

Table S3: Growth yields (log nb cell/ml) of wild-type *T. barophilus* (WT), TbΔMPGP and TbΔMGPS mutants at 24h as a function of hydrostatic pressure, salinity and temperature. NA, not analyzed.

Growth conditions	0.1 MPa			40 MPa			70 MPa		
	1%	3%	4%	1%	3%	4%	1%	3%	4%
80°C	0.7	1.2	0.7	1.8	1.0	0.5	0.6	1.0	0.3
85°C	0.2	1.0	0.1	0.9	**1.0**	0.4	0.2	1.1	0.2
90°C	0.7	0.8	0.4	0.5	0.5	1.1	1.2	1.4	0.7

Table S4 : *T. barophilus* intracellular potassium levels measured for all conditions of growth (pressure, temperature and salinity). Values are referenced to optimal growth conditions, in bold (40 MPa/85°C/3% NaCl), 201.6 mM K^{+}.

**Gene deletions**				
TERMP_00005	MPGS	MPGS-Up	Forward	GAATTTGCAATGAAGCGCTGATCTTTTCT
	MGPS	MPGS-Do	Reverse	AATTCTCAGCGATACGTCAATCATTGTGATA
TERMP_00024	MPGP	MPGP-Up	Forward	CAATCTGCCATTCACGAAGCTGC
	MPGP	MPGP-Do	Reverse	TTCGGTTTTGAGAAGAGCAGCGC

Table S5 : Primers used for gene deletion confirmations.

RT-PCR

gene tag	gene	primer	orientation	forward	gene position
TERMP_00005	MGPS	5F	Forward	TGGACACCGAGACAGAGGAAGTACC	77-101
		5R	Reverse	TCCATCTGAAGCCAAGCTCGAGT	1020-998
TERMP_00024	MPGP	24F	Forward	CAAAAACAAGGGCAGAGCAGGAGTA	122-146
		24R	Reverse	GCCTCTGGATGTTTTAAGTTCCCGA	692-668
TERMP_00025	PMI	25F	Forward	ACGTTCTCCTTGAGCCGGTTGG	236-257
		25R	Reverse	ACGACCCAGTGCTCCGACCT	1181-1162
TERMP_00026	PMM	26F	Forward	TGGGTTGTCGTGGGAAGGGACA	157-178
		26R	Reverse	CTTCGCTCCTCGCCTCTGCG	1339-1320
16S rRNA		Arch344F	Forward	ACGGGGYGCAGCAGGCGCGA	[7]
		Arch910R	Reverse	GCTCCCCCGCCAATTC	[8]

Table S6 : Primers used for RT-PCR amplification and gene deletion confirmations.

Growth conditions	Transcript level of MG synthesis genes			
	MGPS	MPGP	PMI	PMM
85°C	1.0	1.0	1.0	1.0
90°C	1.0	1.0	2.0	3.8
98°C	1.5	6.8	8.4	2.2

Table S7 : Quantitative PCR of cDNA genes synthesis of mannosylglycerate (MG) and di-*myo*-inositol phosphate (DIP) normalized to *16S rRNA* genes. mRNA levels of MG and DIP genes synthesis are referenced to mRNA levels for the optimal growth conditions (optimal P, T and S, i.e. 40MPa/85°C/3% NaCl). The trend was confirmed in duplicate experiments. Growth conditions : 90°C, 0,1 MPa and 4% NaCl; 98°C, 40 MPa and 3% NaCl. ND, not detected.

Literature

1 Chomczynski, P. & Sacchi, N. The single-step method of RNA isolation by acid guanidinium thiocyanate-phenol-chloroform extraction: twenty-something years on. *Nat. Protocols* **1**, 581-585 (2006).

2 Voorhorst, W. G. B., Rik, I. L., Luesink, E. J. & Devos, W. M. Characterization of the *cel*B gene coding for beta-glucosidase from the hyperthermophilic archaeon *Pyrococcus furiosus* and its expression and site-directed mutation in *Escherichia coli*. *J. Bacteriol.* **177**, 7105-7111 (1995).

3 Vannier, P., Marteinsson, V., Fridjonsson, O., Oger, P. & Jebbar, M. Complete genome sequence of the hyperthermophilic piezophilic, heterotrophic and carboxydotrophic archaeon *Thermococcus barophilus* MP. *J. Bacteriol.* **193**, 1481–1482 (2011).

4 Marteinsson, V. T. *et al. Thermococcus barophilus* sp. nov., a new barophilic and hyperthermophilic archaeon isolated under high hydrostatic pressure from a deep-sea hydrothermal vent. *Int. J. Syst. Bacteriol.* **49**, 351-359 (1999).

5 Cario, A., Mizgier, A., Thiel, A., Jebbar, M. & Oger, P. Restoration of the di-myo-inositol-phosphate pathway in the piezo-hyperthermophilic archaeon *Thermococcus barophilus*. *Biochimie* **118**, 288-293 (2015).

6 Zeng, S. *et al. Pyrococcus* CH1, an obligate piezophilic hyperthermophile : extending the upper pressure-temperature limits for life. *ISME J.* **3**, 873-876 (2009).

7 Raskin, L., Stromley, J. M., Rittmann, B. E. & Stahl, D. A. Group-specific 16S rRNA hybridization probes to describe natural communities of methanogens. *Appl. Environ. Microbiol.* **60**, 1232-1240 (1994).

8 Großkopf, R., Janssen, P. H. & Liesack, W. Diversity and structure of the methanogenic community in anoxic rice paddy soil microcosms as examined by cultivation and direct 16S rRNA gene sequence retrieval. *Appl. Environ. Microbiol.* **64**, 960-969 (1998).

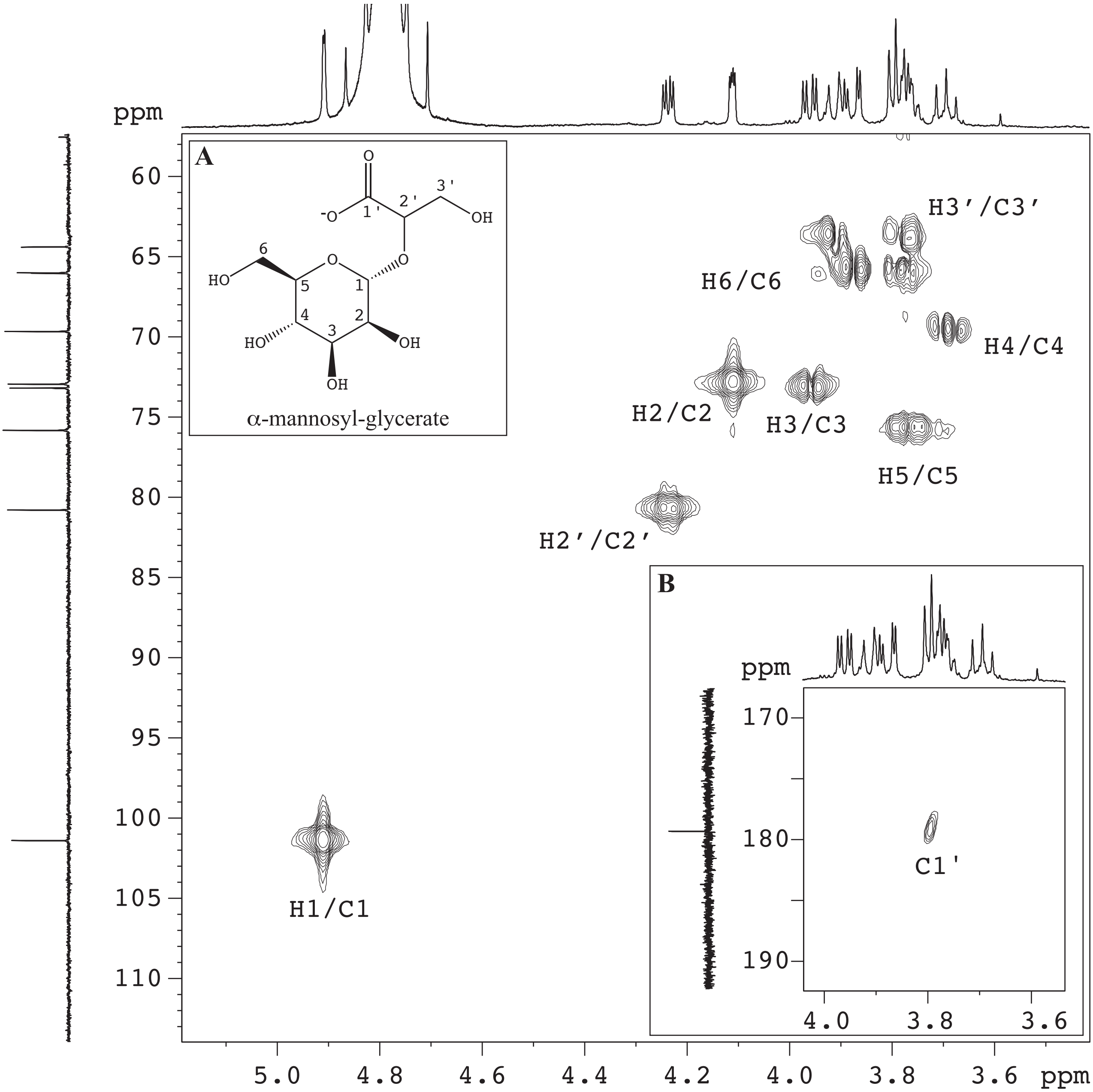

Figure S1 : 13C-1H HMQC of partially purified osmolytes from T. barophilus grown under atmospheric pressure, under salt stress (4%) and optimal temperature (85°C). The specific assignments of MG are reported on the 1H-13C HMQC spectrum. Cross peaks correspond to connectivities between proton and carbon atoms covalently bonded. Insert A : mannosyl glycerate. Numerals represent proton numbering. Insert B : Detail of the C1' region. The chemical shifts for mannosylglycerate are (in ppm for the 13C/1H) 101.4/4.91, 72.96/4.11, 73,2/3.96, 69.67/3.69, 75.84/3.76 and 65,91/(378/3.88) for centers 1, 2, 3, 4 , 5 and 6 of the mannose moiety respectively, and 179.85/-, 80.81/4.24 and 63.85/(3.78/3.93) for the 1', 2' and 3' centers of the glycerate moiety.
